# Systems Analysis of Chaperone Networks in the Malarial Parasite Plasmodium falciparum

**DOI:** 10.1371/journal.pcbi.0030168

**Published:** 2007-09-14

**Authors:** Soundara Raghavan Pavithra, Ranjit Kumar, Utpal Tatu

**Affiliations:** Department of Biochemistry, Indian Institute of Science, Bangalore, Karnataka, India; University of California San Diego, United States of America

## Abstract

Molecular chaperones participate in the maintenance of cellular protein homeostasis, cell growth and differentiation, signal transduction, and development. Although a vast body of information is available regarding individual chaperones, few studies have attempted a systems level analysis of chaperone function. In this paper, we have constructed a chaperone interaction network for the malarial parasite, Plasmodium falciparum. P. falciparum is responsible for several million deaths every year, and understanding the biology of the parasite is a top priority. The parasite regularly experiences heat shock as part of its life cycle, and chaperones have often been implicated in parasite survival and growth. To better understand the participation of chaperones in cellular processes, we created a parasite chaperone network by combining experimental interactome data with in silico analysis. We used interolog mapping to predict protein–protein interactions for parasite chaperones based on the interactions of corresponding human chaperones. This data was then combined with information derived from existing high-throughput yeast two-hybrid assays. Analysis of the network reveals the broad range of functions regulated by chaperones. The network predicts involvement of chaperones in chromatin remodeling, protein trafficking, and cytoadherence. Importantly, it allows us to make predictions regarding the functions of hypothetical proteins based on their interactions. It allows us to make specific predictions about Hsp70–Hsp40 interactions in the parasite and assign functions to members of the Hsp90 and Hsp100 families. Analysis of the network provides a rational basis for the anti-malarial activity of geldanamycin, a well-known Hsp90 inhibitor. Finally, analysis of the network provides a theoretical basis for further experiments designed toward understanding the involvement of this important class of molecules in parasite biology.

## Author Summary

Among the infectious diseases affecting humans, the malarial parasite is responsible for a very high number of deaths. About 2 million people die of malaria every year, of which more than a million are children in sub-Saharan Africa. Malaria is caused by a protozoan parasite belonging to the genus Plasmodium, and Plasmodium falciparum is responsible for the most severe form of malaria. Due to the increasing incidence of resistance to existing drugs, there is a growing need to discover new and more effective drugs against malaria. We have computationally predicted processes governing parasite growth in humans. Recent reports from several labs point to a critical role played by a group of proteins termed molecular chaperones in parasite growth within red cells. We have therefore chosen to provide a balance sheet of all the activities supported by this group of proteins in the parasite. Our systems-level approach provides information on 95 different chaperones in the parasite and also provides insights into their business partners and cellular processes that they might regulate. In addition to predicting a basis for the anti-malarial potential of known drugs such as geldanamycin, our analysis also highlights new proteins that can be used as anti-malarial targets.

## Introduction

Molecular chaperones have emerged in recent years as key players in several cellular processes. In addition to assisting in the folding of newly synthesized proteins, they play crucial roles in the translocation of proteins across organelle membranes, quality control in the endoplasmic reticulum (ER), turnover of misfolded proteins, as well as signal transduction [1–3]. By chaperoning key signaling proteins such as protein kinases and transcription factors, chaperones such as Hsp90 (heat shock protein 90) also participate in cell growth, differentiation, apoptosis, and development [4,5]. As such, many chaperones are also essential under non-stress conditions and are relatively abundant in the cell. There are different families of stress proteins, the major ones being the Hsp40, Hsp60, Hsp70, Hsp90, Hsp100, TCP-1/CCT, and the small Hsp families [6]. These families of chaperones are ubiquitous and conserved in most organisms from bacteria to higher eukaryotes. In recent years, the importance of chaperones in developmental processes has been highlighted by the demonstration of a role for Hsp90 in buffering genetic variation [7–9].

Despite the important processes in which chaperones have been implicated, most studies have been restricted to the analysis of individual chaperones. There is growing realization that chaperones function in complexes with partner co-chaperones and other effectors [10,11]. These modular complexes participate in multiple pathways in the cell, emphasizing the need to examine protein–protein interaction networks for a better understanding of cellular processes. Chaperone interaction networks, for example, may provide a theoretical framework to study the response of a cell to stressful conditions. Keeping in mind the central importance of chaperones in biological processes, we have made an effort at analyzing chaperone interaction networks in human beings, as well as one of the most important human pathogens, the malarial parasite P. falciparum.

During its life cycle, the parasite continually cycles between a cold-blooded insect vector and a warm-blooded human host and experiences episodes of heat shock frequently [12]. Within the human body, the parasite experiences heat shock periodically during the febrile episodes that are a clinical hallmark of malaria. It is therefore possible that heat shock proteins have a role to play in the adaptation to and survival of the parasite in the human host. The malarial parasite expresses several chaperones including proteins of the Hsp40, Hsp60, Hsp70, and Hsp90 families [13–17]. Parasite Hsp70, for example, has been shown to be capable of suppressing the thermosensitivity of a DnaK (Hsp70) mutant of E. coli, suggesting that PfHsp70 may have a cytoprotective role in enabling thermotolerance in the parasite [18]. PfHsp90 has been shown to be essential for parasite viability [17,19], while an Hsp40-like protein (RESA) has been demonstrated to have a role in resistance to heat shock during febrile episodes [20]. Furthermore, heat shock proteins of the erythrocyte cytoplasm such as host-Hsp70 and host-Hsp90 have been demonstrated to participate in parasite-directed remodeling of the host erythrocyte surface [21]. However, there is no study that integrates information about parasite chaperones on a global scale. Such integration is essential if data regarding individual chaperones is to be translated into information that can be used to understand the biology of the parasite.

Protein interaction networks can be determined experimentally through various high throughput methods such as yeast two-hybrid assays and mass spectrometry [22]. An alternative computational approach to determining interaction networks involves the use of interolog analysis [23,24]. A tentative view of the potential protein–protein interaction network in P. falciparum has been provided by the work of LaCount et al. [25]*.* In addition to providing a global view of the protein interaction network in the parasite, comparison of the parasite interaction network with that of the yeast Saccharomyces cerevisiae suggests that the protein network of the parasite may exhibit interesting functional differences worthy of further investigation [26]. While this study has provided the first view of the parasite protein interaction network, the significance of many of the protein–protein interactions has not been discussed in full detail.

One of the computational approaches to deriving protein interaction networks is the interolog method. Interologs are protein–protein interactions that are conserved across species. The interolog approach makes use of the logic that if two proteins are known to interact in one organism, their orthologs in other organisms may also be assumed to be interacting proteins. This approach has been used to predict protein interaction networks in Caenorhabditis elegans, Candida albicans, Drosophila melanogaster, and Arabidopsis thaliana [27].

In this paper, we have constructed a protein interaction network for chaperones from the malarial parasite P. falciparum. We retrieved interaction data from the human protein reference database Human Protein Reference Database (HPRD) (http://www.hprd.org) [28] to create a chaperone interaction network for human cells. We then identified parasite orthologs for the proteins in the human chaperone network by BLASTP analysis and arranged them into protein–protein interactions based on the interactions of the corresponding human proteins. This data was integrated with the interaction data from the yeast two-hybrid screens discussed above [25] to construct a chaperone network for P. falciparum. The uses of such a chaperone network are manifold. In addition to providing clues to the cellular functions governed by parasite heat shock proteins, this network provides a theoretical platform for designing further experiments to elucidate the functions of molecular chaperones during the parasite life cycle.

The chaperone network presented in this paper provides the first systems-level view of chaperone function in the clinically important human pathogen P. falciparum. The network allows us to make testable predictions regarding chaperone function in the parasite. Examination of the network allows us to assign putative functions to chaperones that have not been characterized previously. It also enables us to predict the putative pathways derailed in cells treated with inhibitors of parasite heat shock proteins. An unanswered question in parasite heat shock protein research concerns the identification of the developmental stages during which chaperone function is most crucial. While this question has been difficult to answer experimentally, comparison of chaperone expression levels as well as network parameters during the mosquito stage with the human intra-erythrocytic stages suggests that heat shock proteins may have a role to play during adaptation of the parasite to the human host.

## Results

### List of Chaperones in P. falciparum

Analysis of the P. falciparum genome sequence reveals the presence of genes from all major chaperone families, forming a total of 95 chaperones (see Methods). Table 1 presents the list of chaperones with their PlasmoDB accession numbers grouped according to their families (the list of chaperones along with their transcript and protein abundances is provided in Dataset S1). Certain features of the chaperone families in P. falciparum are of interest. Notably, orthologs of the ER chaperones calnexin and calreticulin are absent from the parasite genome (see Results section for further discussion). The other major difference is the presence of only a couple of genes for small heat shock proteins in the parasite (MAL8P1.78 and PF13_0021). By contrast, the human genome contains several genes for small heat shock proteins [29]. Interestingly, both the Hsp70 and Hsp90 families in the parasite contain only one protein with the EEVD motif characteristic of cytosolic members of these families (PF08_0054 for Hsp70 and PF07_0029 for Hsp90). In contrast, both S. cerevisiae and Homo sapiens contain two cytosolic isoforms (inducible and constitutive) of Hsp70 and Hsp90.

**Table 1:**
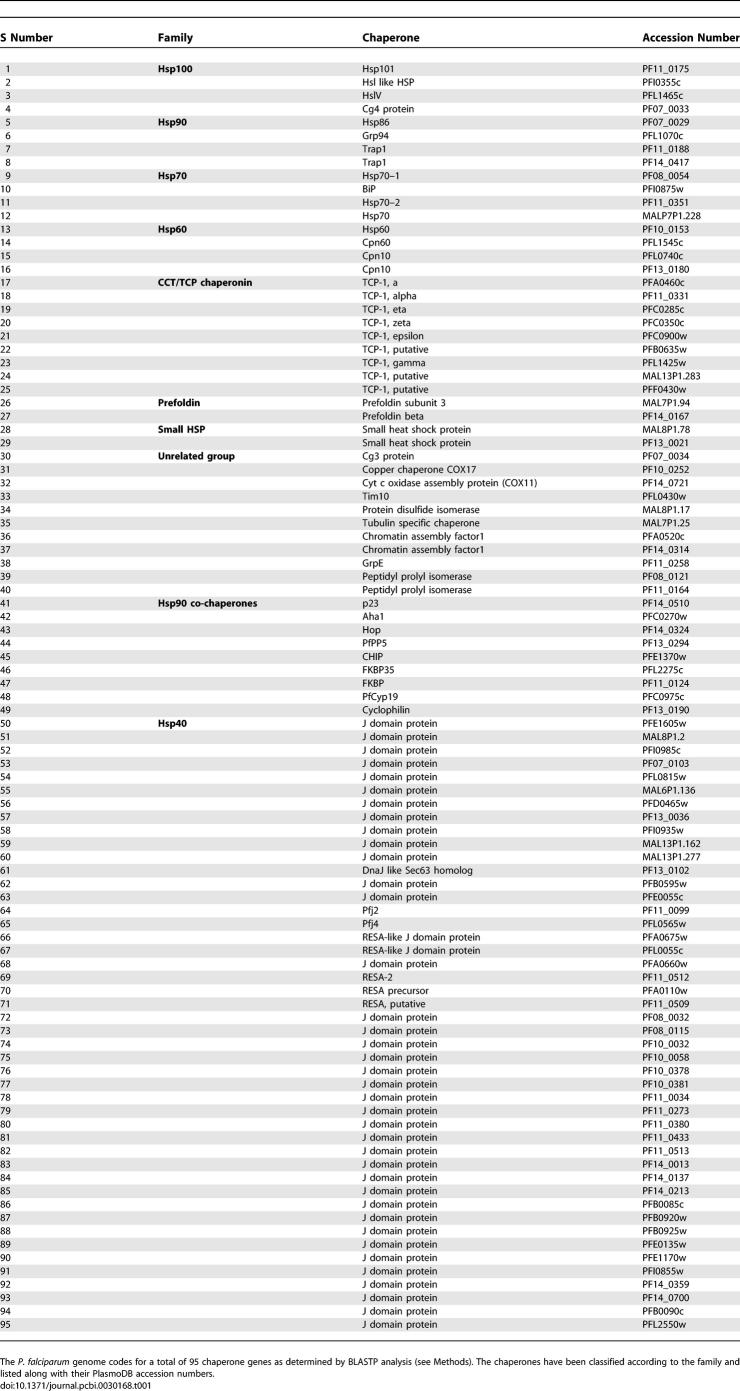
List of Chaperones in P. falciparum

### Human Chaperone Network

As a first step toward constructing a chaperone network in P. falciparum, we created a human chaperone network and then looked for interologs in the parasite. The list of chaperones and their interacting proteins was obtained from HPRD (http://www.hprd.org) and arranged into a network using the software Cytoscape as described in Methods (the list of human chaperones and their interactions is presented in Dataset S2). The human chaperone network is presented in Figure S1 with the interacting proteins color-coded according to their functions. We carried out BLASTP analyses to identify orthologs of the chaperones and their interactors in P. falciparum (see Methods). Those proteins in the human chaperone network (Figure S1) with orthologs in the parasite are indicated in boxes (the list of P. falciparum orthologs of the human chaperones and their interactors is presented as Dataset S3).

The human chaperone network served as a template with which we derived interologs for chaperone–protein interaction in the parasite. Information contained in HPRD is literature-curated, and our network presents the available information on human chaperones and their interacting proteins in a network format. While this does not add new information to the available data, it collates the available information on human chaperones and provides the data in an easy format for researchers working on human chaperone function. The information on human chaperones and their interacting proteins (as a Microsoft Excel file as well as network format) presented in this paper has been incorporated at http://utlab3.biochem.iisc.ernet.in/systems.htm, which will be updated regularly.

### Parasite Chaperone Network

The parasite chaperone network was constructed by combining information obtained from the interolog analysis with that derived from existing high throughput yeast two-hybrid assays. Parasite orthologs of the human chaperones and their interacting proteins obtained from HPRD were identified by BLASTP analyses as described in methods. Protein–protein interactions were predicted for these parasite proteins based on the interactions of the corresponding human orthologs. This interolog network was then combined with the network constructed from the protein–protein interactions determined from the yeast two-hybrid screens of [25] as described in Methods. We also removed those interactions where the two interacting proteins are not co-expressed during any of the various parasite stages, and the final list of protein–protein interactions for parasite chaperones is presented in Dataset S4. The entire dataset was then assembled into a parasite chaperone network with the green lines indicating interactions derived from the yeast two-hybrid data and the red lines indicating interactions predicted by interolog analysis (see Figure S2 for a high-resolution image of the parasite chaperone network).

### Inferences from the Parasite Chaperone Network

#### Prediction of putative functions for hypothetical proteins.

**Figure 1:**
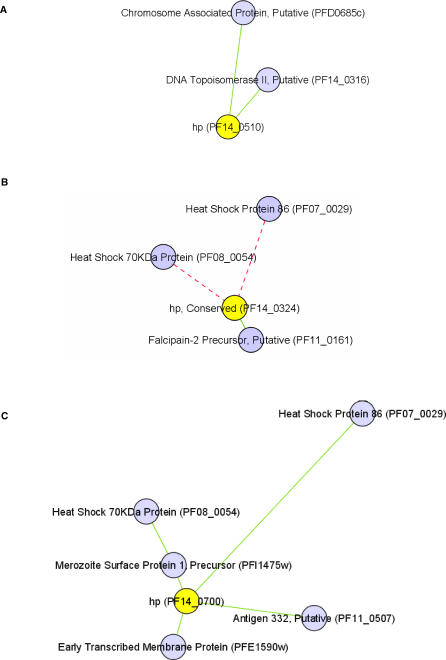
Prediction of Functions for Hypothetical Proteins (A) Involvement of PF14_0510 in transcriptional regulation. (B) PF14_0324 may be involved in haemoglobin metabolism. (C) Participation of PF14_0700 in membrane protein trafficking. The node for which the function is being predicted is highlighted in yellow in each case.

One of the major advantages of constructing protein interactions networks is the ability to predict functions for hypothetical proteins based on their association with functionally characterized proteins. This becomes particularly important in the malarial parasite where 60% of the genome remains un-annotated. We have examined the parasite chaperone network presented here to determine whether we can tentatively identify functions for hypothetical proteins based on the proteins interactions they exhibit. We present our results as follows. First, PFI0335w is a hypothetical protein that interacts with α-tubulin (PFI0180w) in our network. α-tubulin, in turn, shows extensive interactions with TCP-1 chaperonin subunits and actin (PFL2215w) (see Figure S2). It is well-known that in eukaryotes, actin and tubulin folding and dimerization are chaperoned by the TCP-1 chaperonin system in conjunction with a set of tubulin folding co-factors A–E [30]. Examination of the sequence of PFI0335w reveals homology to mouse tubulin folding co-factor B (39% identity with an E value of 3.3 × 10^9^ for PDB entry 1WHG_A). The homology of PFI0335w to tubulin folding cofactor B along with its association with the TCP-1 chaperonin network suggests that PFI0335w may be involved in chaperoning microtubule formation in the parasite. Second, another hypothetical protein PF14_0510 shows homology to the p23 co-chaperone of Hsp90. Interestingly, this p23 ortholog interacts with a number of proteins involved in DNA metabolism. For example, PF14_0510 (henceforth called Pfp23) interacts with DNA topoisomerase II (PF14_0316) and chromosome associated protein PFD0685c (see Figure 1A). These interactions become highly relevant in light of the fact that eukaryotic p23 has been shown to be involved in the disassembly of transcriptional regulatory complexes [31]. Based on the above analysis, it is probable that Pfp23 may function in regulation of transcription within the parasite. Third, PF10_0242 is a hypothetical protein that interacts with parasite heat shock protein 90 (PF07_0029) in the network (see Figure S2). Interestingly, PF10_0242 has an N-terminal domain that shows similarity to MsbA, a PgP-like multi-drug resistance ABC transporter. Human Hsp90β has been shown to interact with PgP and increase drug resistance mediated by PgP [32]. This agrees well with the recent finding that Hsp90 potentiates drug development in pathogenic fungi [33]. Taken together, these results suggest that PF10_0242 may be a PgP-like ABC transporter plausibly involved in drug resistance (see later section for further evidence of link between PfHsp90 and drug resistance). Fourth, PF14_0324 is a hypothetical protein that consists of two TPR domains and exhibits homology to human Hop, a protein that acts as an adaptor linking human Hsp90 and Hsp70 into a multi-chaperone complex (eukaryotic Hsp90 functions as a dynamic multi-chaperone complex; see [4]). Our network accurately predicts interaction of PF14_0324 with PfHsp90 (PF07_0029) and PfHsp70 (PF08_0054), suggesting that PF14_0324 may, indeed, function as an Hsp90-Hsp70 co-chaperone in the parasite. Interestingly, it also interacts with falcipain-2 precursor (PF11_0161), a cysteine protease involved in hemoglobin metabolism within the food vacuole [34] (Figure 1B). An earlier study has demonstrated association of Hsp90 and Hsp70 with ferriprotoporphyrin IX, a toxic product of hemoglobin digestion in the parasite [35]. Taken together, these observations suggest that Hsp90 and Hsp70 may be involved in heme metabolism within the parasite. Fifth, PF14_0700 is a hypothetical protein with a J domain, suggesting that it is a member of the Hsp40 class of chaperones. As shown in Figure 1C, it interacts with PfHsp70 (PF08_0054) indirectly and Hsp90 (PF07_0029) directly, suggesting that it might function as a co-chaperone in the Hsp90 multi-chaperone complex of the parasite. Hsp40 molecules are known to recruit Hsp70 and Hsp90 to particular pathways/clients within the cell [36]. Examination of the interactions exhibited by PF14_0700 indicates that it interacts with a number of membrane/exported parasite proteins, notably early transcribed membrane protein (PFE1590w), antigen 332 (PF11_0507), and merozoite surface protein1 precursor (PFI1475w). Recent studies have suggested a role for parasite Hsp70 in protein trafficking in the parasite [37]. In eukaryotes, Hsp70 has been known to function in concert with Hsp90 in protein trafficking [38,39]. Our analysis, therefore, suggests that PF14_0700 is an Hsp40 molecule that may function to direct parasite Hsp70 and Hsp90 to trafficking of membrane or exported proteins.

#### Chaperones involved in parasite specific pathways.

Examination of the parasite chaperone network in Figure S2 reveals that there is very little overlap between the yeast two-hybrid and interolog interaction subnetworks. While it is difficult to justify the obvious separation of the yeast two-hybrid and interolog datasets, a couple of possibilities do exist. First, it is possible that the non-overlapping nature of the experimentally determined and computationally inferred interactions results from a genuine separation of parasite-specific pathways and evolutionarily conserved ones. This is further substantiated by the observation that several yeast two-hybrid interactions occur between proteins performing parasite specific functions [25]. While it is possible that the observed lack of overlap between the two datasets may arise from a high rate of false positives in the yeast two-hybrid datasets, it may also be a unique feature of the parasite.

We examined the parasite chaperone network for evidence of such separation between various chaperones. Indeed, we find that several Hsp40 chaperones (PFE1605w, PF13_0102, PFL0815w, and PF14_0700), an Hsp90 ortholog (PF11_0188), and an Hsp70 molecule (PF11_0351) exhibit predominantly yeast two-hybrid interactions with few, if any, interolog interactions. In keeping with the argument expressed above, this would suggest involvement of these chaperones in unique parasite-specific pathways. This also agrees well with the observation that the Hsp40 family of chaperones is expanded in the parasite and may participate in cellular processes specific to the parasite [40]. If so, the above chaperones and their associated proteins/pathways may provide a rich source of chemotherapeutic targets for anti-malarial intervention.

#### Changes in the chaperone network during parasite development.

**Figure 2:**
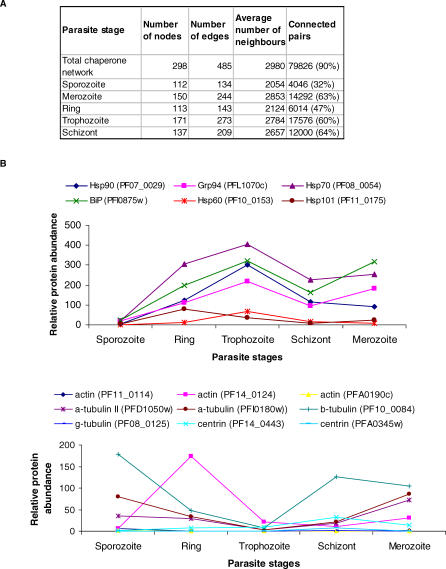
Chaperone Function during Parasite Development (A) Numerical comparison of the various stage-specific chaperone networks. (B) Top panel. Quantitation of the amounts of the major parasite heat shock proteins in various parasite stages. Bottom panel. Quantitation of the abundance of actin, tubulin, and centrin proteins in the different stages.

As part of the life cycle of the parasite, sporozoite stages from the mosquito are injected into humans where they grow chiefly within the red blood cells after a brief sojourn in the liver. During transmission from insects with a body temperature of ∼25 °C [41] to human beings with a body temperature of 37 °C, the parasite experiences a severe heat shock. However, it has been experimentally difficult to determine whether heat shock proteins play a crucial function in parasite adaptation to such a change in the environment. We constructed stage-specific networks containing only those chaperones and interacting proteins expressed in a specific stage (figures for the stage-specific networks are presented in high-resolution format in Figure S3A–S3E). Figure 2A shows a numerical comparison of the sporozoite-specific chaperone network with that for the intra-erythrocytic stages. The data shows that the number of nodes and edges is greater for the intra-erythrocytic stages compared with the sporozoite stage. Likewise, the average number of neighbours as well as connected pairs is greater for the red blood cell stages versus the sporozoite stage. While the above data needs to be interpreted with caution, it suggests that chaperone function may become crucial upon entry of the parasite into the human body.

We also obtained the expression levels of parasite chaperones as well as their interacting proteins from existing experimental data [42] and compared the abundance of chaperones with that of a control group of proteins for different parasite stages. Figure 2B (top panel) shows the quantitation of the amounts of the major chaperones in different stages, indicating that their expression levels are higher in the red blood cell stages compared with the sporozoite stage. As control, we examined actin, tubulin, and centrin expression levels in the same parasite stages. As shown in Figure 2B (bottom panel), the abundance of these proteins does not reveal increased expression in the intra-erythrocytic stages (see Dataset S5 for relative abundances of chaperones and control proteins). These results suggest that heat shock proteins may be induced during transformation of the parasite from the sporozoite stage in the mosquito vector to the intra-erythrocytic stages in the human host. Furthermore, experimental evidence indicates that a heat shock is essential for transformation of sporozoites into the liver stages of the parasite and that parasite hsp70 is induced during this process [43]. The data on the expression levels taken together with the tentative network analysis suggests that heat shock proteins are induced during transmission from mosquitoes to human beings and help in the development or adaptation of the parasite to its new environment.

### Putative Mechanism for the Anti-Malarial Activity of the Hsp90 Inhibitor Geldanamycin

**Figure 3:**
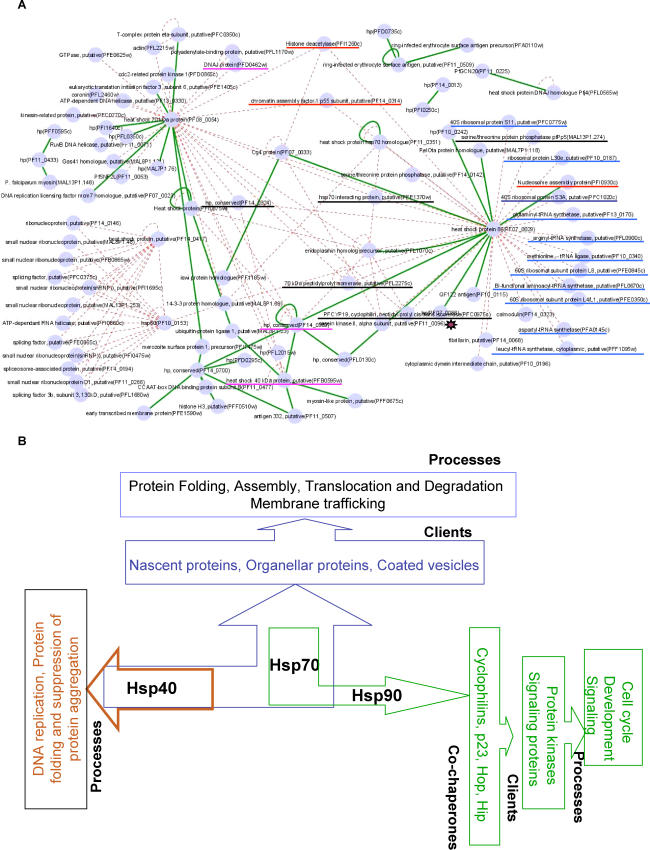
Network of PfHsp90 and PfHsp70 Interactions (A) Subnetwork of PfHsp90 (PF07_0029) and PfHsp70 interactions. Green lines indicate protein–protein interactions derived from yeast two-hybrid screens, while the red lines indicate interactions predicted through interolog analysis. PfHsp90 client proteins indicating involvement of PfHsp90 in particular cellular processes are underlined by blue and red lines. The black lines indicate PfHsp90 co-chaperones while the star indicates casein kinase II alpha subunit, a protein that may be involved in the phosphorylation of PfHsp90. The boxed protein is Cg4, a PfHsp90 interactor that may be associated with drug resistance while the pink lines mark the three Hsp40 proteins that may function as PfHsp70 co-chaperones. (B) Schematic representation of the involvement of Hsp70, Hsp90, and Hsp40 in the biological processes of a typical eukaryotic cell.

Resistance to conventional anti-malarial drugs such as chloroquine and pyrimethamine-sulfadoxine has resulted in an increasing need to identify new chemotherapeutic targets in malaria [44–46]. A drug that targets a component involved in several pathways simultaneously stands less chance of being rendered useless by resistance mechanisms. The Hsp90 homolog of the parasite (PF07_0029) makes such an ideal candidate. Geldanamycin (GA), a member of the benzoquinone ansamycin class of drugs, binds to Hsp90 and inhibits its chaperone activity [47,48]. In fact, GA has been employed as a pharmacological probe of Hsp90 function, and one of its derivatives is currently in clinical trials as an anti-tumor agent [49,50]. However, the precise mechanism by which interference with Hsp90 activity leads to parasite growth inhibition is not clear. Examination of the parasite chaperone network may provide tentative clues by providing a list of client proteins and pathways regulated by PfHsp90. Analysis of the PfHsp90 interaction network in Figure 3A (see Figure S4 for high resolution image) reveals that parasite Hsp90 chaperones proteins functioning in diverse cellular processes. Parasite Hsp90 appears to chaperone proteins involved in translational processes such as bi-functional aminoacyl-tRNA synthetase (PFL0670c), aspartyl-tRNA synthetase (PFA0145c), glutaminyl-tRNA synthetase (PF13_0170), leucyl-tRNA synthetase (PFF1095w), arginyl-tRNA synthetase (PFL0900c), ribosomal proteins S3A (PFC1020c), S11 (PFC0775w), L4/L1 (PFE0350c), L8 (PFE0845c), and L30 (PF10_0187), and methionine-tRNA ligase (PF10_0340) (see proteins underlined by blue lines in Figure 3A). Its interactions with chromatin assembly factor 1 p55 subunit (PF14_0314), nucleosome assembly protein (PFI0930c), and histone deacetylase (PFI1260c) suggest a role for PfHsp90 in chromatin remodeling in the parasite (see proteins underlined by red lines in Figure 3A). In all, our analysis of the chaperone network suggests an important role for PfHsp90 in cellular processes such as chromatin remodeling and protein translation.

The accuracy of the parasite chaperone network is validated by the fact that the network predicts interactions between Hsp90 and a host of proteins that are orthologs of human Hsp90 co-chaperones. Specifically, the network predicts interaction of PfHsp90 with PfHsp70 (PF08_0054), PfPP5 (MAL13P1.274), PfFKBP35 (PFL2275c), PfCyp19 (PFC0975c), PfCHIP (PFE1370w; although annotated as a hypothetical protein in the PlasmoDB database, it shows homology to human CHIP), and PfHop (PF14_0324; although annotated as a hypothetical protein, BLASTP analysis reveals homology to human and yeast Hop proteins) (see proteins underlined by black lines in Figure 3A). Of these, the interactions between PfHsp90, PfHsp70, and PfPP5 have been confirmed by co-immunoprecipitation experiments performed in our laboratory [51], while the interaction between PfHsp90 and PfFKBP35 has been demonstrated by another group [52]. These serve to validate the predictions of our network.

Eukaryotic Hsp90 chaperone activity is regulated by several post-translational modifications, of which phosphorylation is the best-studied [53]. The protein kinase Casein kinase II has been shown to phosphorylate Hsp90 as well as its co-chaperone Cdc37 and thereby modulate its chaperoning of client proteins [54,55]. Our network reveals interaction of PfHsp90 with parasite casein kinase II alpha subunit (PF11_0096), suggesting that phosphorylation of PfHsp90 may form a means of regulating its activity (see protein indicated by a star in Figure 3A). This is further substantiated by experiments from our laboratory that have revealed in vivo phosphorylation of PfHsp90 during the intra-erythrocytic cycle [17].

An important outcome of constructing chaperone networks is the ability to propose testable hypotheses regarding the system under consideration. The above analysis of PfHsp90 interacting proteins suggests that this molecular chaperone plays a central role in cellular processes. It is reasonable, therefore, to assume that the parasite critically depends on PfHsp90 function. In fact, treatment of parasite cultures with GA has been shown to halt stage development during the intra-erythrocytic cycle of the parasite, suggesting that PfHsp90 is essential for parasite development within red blood cells [17,56]. However, the exact mechanism by which Hsp90 regulates development in the parasite is not clear. The chaperone network provides a tentative list of PfHsp90 interacting proteins and the processes it regulates, allowing us to further explore the potential of this protein as a much-needed chemotherapeutic target against malaria.

### Predictions on the PfHsp40 Family

Hsp40 proteins are Hsp70 co-chaperones that modulate interaction of Hsp70 molecules with client proteins by different mechanisms: a) Hsp40s bind and deliver specific client proteins to Hsp70; b) Hsp40 molecules stimulate the ATPase activity of Hsp70 and stabilize Hsp70-client complexes; and c) certain Hsp40s are localized to specific locations within the cell and serve to recruit Hsp70 to these sites [36]. The defining feature of Hsp40 chaperones is the presence of a conserved “J domain” and hence they are also referred to as J domain proteins [57]. P. falciparum codes for several Hsp40 proteins (see list of chaperones in Table 1) and comparison with Escherichia coli and S. cerevisiae indicates that the ratio of Hsp40 genes to total number of genes is higher in the parasite [40]. Taken in conjunction with the presence of a single cytosolic Hsp70 chaperone, this may indicate the presence of new, parasite-specific pathways to which PfHsp70 may be recruited by parasite Hsp40s. This is further corroborated by the fact that several Hsp40 proteins in the network seem to exhibit predominantly yeast two-hybrid interactions (see the earlier section “Chaperones involved in parasite specific pathways”).

Although both Hsp70 and Hsp40 have been characterized in the parasite, the identity of the PfHsp70 interacting Hsp40 co-chaperone(s) is not clear. Analysis of the parasite chaperone network indicates that three Hsp40 proteins (PFB0595w, PFD0462w, and PF14_0359) interact directly with PfHsp70 (PF08_0054) (see proteins underlined by pink lines in Figure 3A). In the absence of information regarding Hsp70–Hsp40 interactions in the parasite, the chaperone network presented here allows us to predict putative Hsp40 co-chaperones for PfHsp70. These three proteins represent Hsp40 co-chaperones that directly interact with Hsp70 and provide a framework for experimental analysis. Figure 3B summarizes the current state of knowledge about Hsp90, Hsp70, and Hsp40 chaperones in a eukaryotic cell in a schematic format. Hsp70 and Hsp40 perform certain functions in concert, while some Hsp40s may also operate independently. Likewise, Hsp70 and Hsp90 also cooperate in certain cellular pathways. The enhanced importance of the Hsp40 family in P. falciparum is highlighted in bold in Figure 3B.

### Insights into the PfHsp100 Family from the Chaperone Network

**Figure 4:**
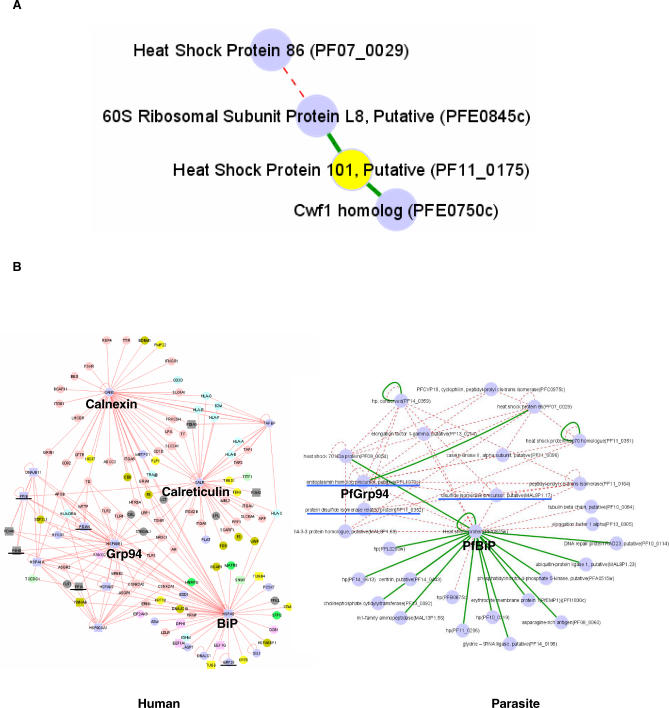
Inferences from Networks Analysis (A) Network of interacting proteins for PfHsp101 (PF11_0175). (B) Left panel. Subnetwork of calnexin, calreticulin, BiP, and Grp94 in the ER of human cells. Underlined proteins indicate interactions between BiP, Grp94, protein disulfide isomerases (P4HB, PDIA4, PDIA6), peptidyl prolyl isomerases (PPIA, PPIB), and ERP29. Right panel. Subnetwork of the parasite ER chaperones, PfBiP and PfGrp94. Proteins indicated by blue lines mark interactions between PfBiP, PfGrp94, peptidyl prolyl isomerase (PF11_0164), and protein disulfide isomerases (MAL8P1.17 and PF11_0352). The protein marked with the red line is PfEMP1 ((PFI1830c).

Hsp100/Clp family members are involved in several processes such as protein degradation, DNA replication, reactivation of aggregated proteins, and inheritance of prion-like factors [58]. These proteins are related to the Hsp70 family but are characterized by significantly larger molecular weights. Although the malarial parasite genome encodes four genes of the Hsp100 family (see list of chaperones in Table 1), these proteins have not been characterized biochemically. Analysis of the parasite chaperone network reveals that an Hsp100 family member (PF11_0175; henceforth called PfHsp101) interacts with the 60S ribosomal protein L8 (PFE0845c) that in turn interacts with PfHsp90 (see Figure 4A). It has been shown that the yeast Hsp100 homolog Sse1p is an Hsp90 co-chaperone and facilitates Hsp90 chaperoning activity [59]. As detailed in the section above, PfHsp90 interacts with several proteins involved in translational processes including the 60S ribosomal subunit protein L8 (PFE0845c). Our network suggests that PfHsp101 may function as a PfHsp90 co-chaperone for protein translation within the parasite. In addition, one of the PfHsp101 interacting proteins (PFE0750c) exhibits homology to Cwf1, a protein that co-purifies with the spliceosome from HeLa cell extracts and has been implicated in pre-mRNA splicing and cell cycle control [60]. This raises the interesting possibility that PfHsp101 may be involved in similar processes within the parasite.

Another Hsp100 family member, the chloroquine resistance–associated gene Cg4 (PF07_0033), was originally identified as a protein linked to chloroquine resistance located in a 36-kb region of Chromosome 7 linked to chloroquine resistance [61]. Our network predicts interaction of Cg4 with PfHsp90 (see boxed protein in Figure 3A). In addition, Hsp90 has recently been shown to potentiate the development of drug resistance in C. albicans in a calcineurin (also known as protein phosphatase PP5)–dependent manner [33]. In this connection, it is important to note that PfHsp90 has been shown to interact with PfPP5 (MAL13P1.274) by co-immunoprecipitation experiments [50]. Taken together with the interaction of PfHsp90 with the chloroquine resistance–associated protein Cg4, the above analysis suggests that PfHsp90 may play a role in the development of drug resistance in the malarial parasite. Although Hsp100 chaperones have not been studied in P. falciparum, the parasite chaperone network presented in this paper allows us to speculate on putative cellular functions performed by these chaperones and provides a theoretical basis for designing experiments to decipher its role in parasite biology. Furthermore, the network also allows the development of a testable hypothesis regarding the putative involvement of PfHsp90 in the evolution of drug resistance in the parasite.

### The Parasite Network Lacks Two Important Nodes

Protein folding within the ER is regulated by molecular chaperones in a process termed “quality control” [62]. Molecular chaperones bind transiently to nascent polypeptide chains and prevent aggregation as well as incorrect assembly of proteins. BiP (ER member of the Hsp70 family), Grp94 (member of Hsp90 family), and calnexin and calreticulin (related chaperones—the former is membrane-bound while the latter is soluble) are some of the most important chaperones in the ER of eukaryotic cells. Calnexin and calreticulin are involved in the chaperoning of N-glycosylated proteins within the ER. However, their role in the chaperoning of non-glycosylated proteins is still controversial [63,64]. Figure 4B presents the subnetwork of the main ER chaperones from humans as well as the malarial parasite. While the human subnetwork contains BiP (HSPA5), Grp94 (HSP90B1), calnexin (CANX), as well as calreticulin (CALR), the parasite network contains only PfBiP (PFI0875w) and PfGrp94 (PFL1070c). The absence of calnexin and calreticulin is in agreement with experimental evidence suggesting that N-glycosylation of proteins is absent in the parasite [65–67]. It is suggestive that in a system where N-glycosylation of proteins is absent, calnexin and calreticulin are missing as well. This suggests that calnexin and calreticulin may not chaperone non-glycosylated proteins and that their absence might not greatly affect the biological processes of the ER in the absence of glycosylation.

While calnexin and calreticulin are absent from the parasite network, other ER chaperones such as peptidyl prolyl isomerases and protein disulfide isomerases are present in the parasite (see list of parasite chaperones in Table 1). It has been shown that the ER of human cells contains a complex of chaperones including BiP and Grp94 that is involved in folding of secretory proteins [68,69]. This can also be seen in the network in Figure 4B (left panel; see underlined proteins) that shows interactions between BiP, Grp94, protein disulfide isomerases (P4HB, PDIA6, PDIA4), peptidyl prolyl isomerases (PPIA, PPIB), and ERP29. The parasite network indicates that such a scenario may occur in the parasite also (note interactions between PfBiP, PfGrp94, peptidyl prolyl isomerase (PF11_0164), and protein disulfide isomerases (MAL8P1.17 and PF11_0352; see proteins underlined in blue in Figure 4B, right panel). Our analysis suggests that parasite ER chaperones also function in concert as a complex in a manner similar to the human ER chaperones.

### Chaperone Involvement in Cytoadherence

An important feature of the malarial parasite, P. falciparum, is its ability to sequester within the host vasculature by adhering to the endothelial cells of the host. This property, called cytoadherence, is responsible for cerebral malaria caused by parasite sequestration within the vasculature of the human brain [70]. The property of cytoadherence is associated with structures called “knobs” on the infected erythrocyte surface that adhere to receptors on the endothelial cells [71]. Following invasion of a red blood cell, the parasite transports certain proteins to the infected erythrocyte surface to form the knob structure. The receptor binding protein in the knob is PfEMP1 (erythrocyte membrane protein 1) [72]. This protein varies in size between different parasite strains and is highly antigenically variable. Interestingly, the network of parasite ER chaperones predicts an interaction between the ER homolog of Hsp70 (PFI0875w; also called PfBiP) and PfEMP1 (PFI1830c) (see protein indicated by a red line in Figure 4B, right panel). Experimental evidence suggests that parasite proteins exported to the erythrocyte compartment such as knob components have a hydrophobic signal near the N-terminus that directs translocation into the ER and default trafficking to the parasitophorous vacuole [73]. Furthermore, PfEMP1 trafficking to the erythrocyte surface has been shown to occur by a complex pathway involving passage via the ER [74]. It is plausible that PfBiP chaperones PfEMP1 during its passage through the ER in a manner similar to the chaperoning of secretory proteins by human BiP. As mentioned in the preceding section, parasite ER chaperones may function together as a part of a complex. It is therefore tempting to speculate that parasite proteins trafficked via the ER are chaperoned by this protein complex. Interfering with this chaperone complex may then provide a potential anti-malarial intervention strategy.

## Discussion

Recent years have seen the accumulation of a huge amount of information regarding the temporal and spatial expression of genes and proteins in the malarial parasite P. falciparum [41]. However, cellular functions result from the interactions of proteins with one another and with the environment at large, making it imperative to construct protein–protein interaction networks. Such networks have been constructed for P. falciparum using experimental as well as computational approaches [25,75]. The advantage of constructing these networks lies in the ability to understand cellular processes at the systems level. In this paper, we have carried out a systems level analysis of chaperone networks in the malarial parasite. We have used a two-pronged approach: a human chaperone network was first constructed using the list of chaperones and their interacting proteins from the HPRD database. We then used the “interolog” concept to identify those parasite proteins that are orthologs of the chaperones and interactors in the human chaperone network. While we have used human chaperones and their interactors to derive interologs in the parasite, prediction of parasite protein–protein interactions could also have been carried out using information from other model organisms, not just human protein–protein interactions. The reason we have used information from the human system is two-fold: first, information from HPRD has been manually extracted from the literature by careful analysis of published data. Second, human beings are the vertebrate host for P. falciparum and comparison with the human chaperone network may aid in the identification of parasite-specific processes chaperoned by heat shock proteins. This, in turn, may facilitate the design of drugs targeting these processes. While the derivation of interologs from other organisms is also possible, it is important to note that it has been shown that the Plasmodium protein network diverges significantly from those of other eukaryotes [26]. A comparison of the protein–protein interaction network of Plasmodium reported by LaCount et al*.* with the protein networks for S. cerevisiae, C. elegans, D. melanogaster, and Helicobacter pylori reveals that Plasmodium has only three conserved complexes with yeast and no conserved complexes with any of the other organisms examined [26]. In light of this data, the lack of interologs from other organisms may not signifcantly reduce the quality of the network. To the interolog dataset, we added data from the yeast two-hybrid analysis of parasite protein–protein interactions [25] to construct a network of chaperone–protein interactions in the parasite.

The network fulfills the following functions. First, it predicts putative functions for several hypothetical proteins in the parasite. Since a major portion of the parasite genome remains un-annotated, an important goal of interactome projects is to tentatively predict functions for these proteins. Our network fulfills this goal and provides several examples where clues to the functions of hypothetical proteins are provided by their interactions. Briefly, we have been able to implicate PFI0335w in the chaperoning of tubulin folding and PF14_0510 in the regulation of transcription. An Hsp40 molecule (PF14_0700) has been implicated in membrane protein trafficking in concert with PfHsp70 and PfHsp90. Our analysis also suggests that PF10_0242 and PF14_0243 may be involved in drug resistance and hemoglobin metabolism within the parasite. Our network, therefore, provides testable hypotheses expected of a systems level analysis of chaperone function in the parasite. Second, it draws attention to classes of chaperones that have not been characterized in the parasite and provides a basic framework for further analysis. For example, while the Hsp100 family has not so far been studied in the parasite, our network provides clues to the involvement of these chaperones in protein translation and drug resistance. In addition, although the parasite Hsp70 and Hsp40 families have both been explored, the interconnectivities between these two chaperone systems have not been elucidated. Our network shows that, out of the 27 intracellular J proteins in the parasite [40], only three may interact with PfHsp70. This narrows the search for Hsp70-interacting DnaJ co-chaperones considerably and facilitates the design of specific experiments to explore the interaction of PfHsp70 with Hsp40 co-chaperones. Third, it highlights chaperone systems that are missing in the malarial parasite. The ER chaperones dedicated toward quality control of glycosylated proteins in eukaryotic cells (calnexin and calreticulin) are lacking in the parasite chaperone network. While the participation of calnexin and calreticulin in the chaperoning of non-glycosylated proteins has been the centre of considerable debate [62,63], the absence of glycosylation in the parasite suggests that calnexin and calreticulin may not be involved in the chaperoning of non-glycosylated proteins. Thus, the network allows us to propose a tentative solution to an ongoing debate regarding ER chaperone function. Fourth, the chaperone network provides a tentative basis for the anti-malarial activity of drugs affecting the chaperone system. While it has been established that GA inhibits parasite development [17,55], the mechanism by which it does so has not been elucidated. The parasite chaperone network provides a list of interacting proteins and processes that might be affected by inhibition of PfHsp90 function and thereby provides a putative mechanism for GA action. In this sense, analysis of the parasite chaperone network underlines the potential of Hsp90 as a chemotherapeutic target. Finally, the network also allows us to determine the functional significance of chaperones during the different developmental stages of the parasite. The life cycle of the parasite involves transition from a cold-blooded insect vector with a body temperature of ∼25 °C to a warm-blooded human host with a body temperature of ∼37 °C. It is logical to assume that the heat shock involved in this process might result in the induction of heat shock proteins which might subsequently help in the adaptation of the parasite to its new environment. However, it has been difficult to test this hypothesis experimentally. Comparison of the interaction parameters and expression levels of parasite chaperones in the mosquito stage (sporozoite) with the intra-erythrocytic stages (ring, trophozoite and schizont, merozoite) provides a tentative clue. The sporozoite stages express low levels of all major heat shock proteins while the red blood cell stages contain considerable levels of the same. A control group of proteins (actin, tubulin, and centrin), on the other hand, do not exhibit increased expression in the red blood cell stages. In addition, the number of nodes, edges, average number of neighbors, and connected partners is also greater for the red blood cell stages compared with the sporozoite stage. While the lack of information on the hepatic and pre-erythrocytic stages prevents us from drawing conclusions about these developmental stages, the above evidence suggests that chaperone function may be more crucial within the vertebrate human host rather than within the invertebrate mosquito vector.

While the network presented in this paper provides valuable clues to chaperone function in the parasite, it is also important to consider the limitations of this study. In our study, we have combined protein–protein interaction data from experimental (yeast two-hybrid assays) and computational (interolog) setups. While this provides better coverage of interaction data, one must also consider the pitfalls of this approach. Yeast two-hybrid assays are not 100% accurate and provide many putative interactions that cannot be confirmed by other experiments (false positives). False negatives (missed interactions) may also occur due to peculiarities in the experimental setup that prevent interaction between two proteins or from problems in the assembly of the two transcriptional domains (activation and DNA binding) needed for yeast two-hybrid assays. As a result, yeast two-hybrid assays do not provide complete coverage [76]. In addition, it must be noted that interactions predicted in silico by interolog mapping are hypothetical until they are validated in the relevant organism. Moreover, our analysis is also limited to those proteins that have been annotated in PlasmoDB, are orthologs of proteins in other organisms, or have been experimentally characterized in the parasite. As a result, a vast number of hypothetical proteins have been ignored in our analysis. It is important to remember, therefore, that the network presented here may not represent the complete set of chaperone–protein interactions in the parasite.

In summary, we have constructed a chaperone network for the malarial parasite P. falciparum. Understanding the biology of this clinically important human pathogen is essential for rational development of anti-malarial drugs. The network presented here provides a broad view of the functions of molecular chaperones in the parasite. Analysis of the parasite chaperone network highlights the functions carried out by parasite chaperones while aiding the development of drugs specific to the parasite.

## Methods

### Construction of human chaperone network.

A list of human chaperones was obtained from HPRD (http://www.hprd.org) [28]. A total of 149 chaperones were retrieved and their protein–protein interactions extracted using home-written PERL scripts from HPRD. This resulted in a final list of 797 proteins (including chaperones and their interactors) involved in 1,382 protein–protein interactions. These interactions were arranged into a network using the program Cytoscape [77]. Proteins in the network were labeled according to HPRD nomenclature and color-coded according to their functions. To create the subnetwork of BiP, Grp94, calnexin, and calreticulin in humans, protein–protein interactions for these were retrieved from the dataset of 1,382 interactions and arranged into a network. The list of 149 human chaperones, 797 chaperones and interactors, as well the list of 1,382 protein–protein interactions is presented in Dataset S2.

### Identification of chaperones in P. falciparum.

We identified a total of 95 chaperones in the parasite by BLASTP analysis. As input, we used the sequences of the human and yeast (S. cerevisiae) counterparts for the Hsp60, Hsp70, Hsp90, Hsp100, and CCT families, as well as the Hsp90 co-chaperones. The prefoldin, small HSP, and the unrelated group of chaperones were retrieved from the PlasmoDB database. The Hsp40 sequences were identified by an NCBI BLAST search using the E. coli DnaJ as a query against the PlasmoDB database with the typical 4-alpha-helical secondary structure of the J-domain as an additional criterion [40]. The transcript and protein abundances for these chaperones were retrieved from published data [30]. The list of parasite chaperones as well as their transcript and protein abundances is presented in Dataset S1.

### Interolog analysis.

To derive potential protein–protein interactions in P. falciparum from the human chaperone network, we used an approach called generalized interologs mapping described in [27]. To identify orthologs of the human chaperones and their interacting proteins in P. falciparum, we carried out BLASTP analysis of the PlasmoDB sequence database (http://www.plasmodb.org) using the sequences of the human proteins obtained from HPRD as input. We then selected those parasite sequences that exhibited an E value < 10^−10^, bit score > 50, and sequence coverage > 50%. We only selected those sequences that showed coverage over the entire length of the sequence. Also, since Plasmodium proteins often have low-complexity regions, we used the default setting of NCBI BLAST 2.0 to filter query sequences for low-complexity regions. Finally, we obtained a list of 201 putative orthologs in P. falciparum. Another five orthologs were added based on a good E value score and their presence in the OrthoMCL database (http://orthomcl.cbil.upenn.edu), resulting in a total list of 206 parasite orthologs of human chaperones and their interactors. We then predicted protein–protein interactions for these 206 parasite orthologs based on the interactions of the corresponding human proteins. The list of 206 parasite orthologs is presented in Dataset S3.

### Construction of the parasite chaperone network.

We constructed the parasite chaperone network by combining two subnetworks. The first network was based on protein–protein interactions derived from the yeast two-hybrid experiments of LaCount et al. [25]. The parasite genome contains a total of 95 chaperone genes as determined by manual BLASTP analysis of the genome for the various families of heat shock proteins and we extracted protein–protein interactions for these from the yeast two-hybrid data. The second network consisted of the interologs obtained from the human chaperone network as described above. The yeast two-hybrid assays provided 224 interactions, while 271 interactions were derived using the interolog approach. Combined together, the two approaches yielded a total of 312 parasite proteins involved in 495 interactions. We then removed those interactions where the partners were not co-expressed in even one of the various parasite stages. This resulted in a final dataset of 344 interactions between 212 proteins presented in Dataset S4. These interactions were arranged into a network using the program Cytoscape [77], with the green lines depicting interactions derived from the yeast two-hybrid data and the red lines indicating interactions predicted by the interolog approach. All parasite proteins were labeled by their PlasmoDB accession numbers. To create a subnetwork of PfHsp90 (PF07_0029) and PfHsp70 (PF08_0054) interactions, we retrieved the data for protein–protein interactions involving these proteins from the total list of parasite protein interactions obtained above and arranged these into a network using Cytoscape. Likewise, to create a subnetwork of the ER chaperones PfBiP (PFI0875w) and PfGrp94 PFL1070c), interactions for these proteins were retrieved and arranged into a network using Cytoscape. To compare the expression levels of chaperones and control proteins, we retrieved their protein abundances from the Le Roch et al. dataset [42] and plotted them in a graph using Microsoft Excel. To construct the stage-specific networks, we retrieved protein–protein interactions for proteins present in a particular stage and arranged them into a network. The list of proteins expressed in a specific stage along with their protein–protein interactions (wherever present) is given in Dataset S6. The number of nodes, edges, average number of neighbors, and connected pairs were determined for each stage-specific network using the Cytoscape network analyzer software [77].

## Supporting Information

Dataset S1 List of Chaperones in P. falciparum along with Their Transcript and Protein Abundances (29 KB XLS)

Dataset S2 List of 149 Human Chaperones, 797 Proteins (Chaperones + Interactors), and 1,382 Protein–Protein Interactions between the 797 Proteins The details of each of these proteins are also included.
(230 KB XLS)

Dataset S3 P. falciparum Orthologs of the Human Chaperones and Interacting Proteins from HPRD Are Listed along with Their E Values, Bit Score, Degree of Identity and Similarity, Gaps, and Sequence Coverage Proteins in green represent those retrieved from the OrthoMCL database due to good E value and identity.
(140 KB XLS)

Dataset S4 The Total List of Proteins in the Parasite Chaperone Network Is Presented along with Their Details The list of yeast two-hybrid and interolog interactions for these proteins is presented along with the total list of protein–protein interactions. pp indicates interolog interactions while py indicates yeast two-hybrid derived interactions.
(140 KB XLS)

Dataset S5 Relative Abundances of Chaperones and Control Proteins (Actin, Tubulin, and Centrin) in Sporozoite, Ring, Trophozoite, Schizont, and Merozoite Stages (21 KB XLS)

Dataset S6 Stage-Specific Networks List of proteins expressed in a specific stage along with their protein–protein interactions (where applicable) for sporozoite, ring, trophozoite, schizont, and merozoite stages.
(94 KB XLS)

Figure S1 Human Chaperone Network Proteins are color-coded according to their functions as follows: gray, enzyme; aqua, immunity; lime, translation; orchid, nucleotide metabolism; blue, protein folding and degradation; hot pink, signal transduction; yellow, structural protein; marine, transcription; orange, miscellaneous. Those proteins that have orthologs in P. falciparum are indicated in boxes.
(1.5 MB PNG)

Figure S2 Parasite Chaperone Network Green lines indicate protein–protein interactions derived from yeast two-hybrid screens while the red lines indicate interactions predicted through interolog analysis.
(2.9 MB PNG)

Figure S3 Stage-Specific Networks Representing Interactions of All Proteins Expressed in a Specific Stage (A) Sporozoite specific network. (B) Ring stage network. (C) Trophozoite stage network. (D) Schizont specific network. (E) Merozoite stage network.
(709 KB PDF)

Figure S4 Subnetwork of PfHsp90 and PfHsp70 Interactions Green lines indicate protein–protein interactions derived from yeast two-hybrid screens, while the red lines indicate interactions predicted through interolog analysis.
(1.0 MB PNG)

## Abbreviations

ER: endoplasmic reticulum
GA: geldanamycin
HPRD: Human Protein Reference Database
Hsp/HSP: heat shock protein
