# Supplementary material for: Systems Analysis of Chaperone Networks in the Malarial Parasite Plasmodium falciparum
Source: PLoS Comput Biol. 2007 Sep 14;3(9):e168. doi: 10.1371/journal.pcbi.0030168 (PMC1976336; doi:10.1371/journal.pcbi.0030168)
Supplement: Supplementary file 1 [file pcbi.0030168.sg003.pdf]

A

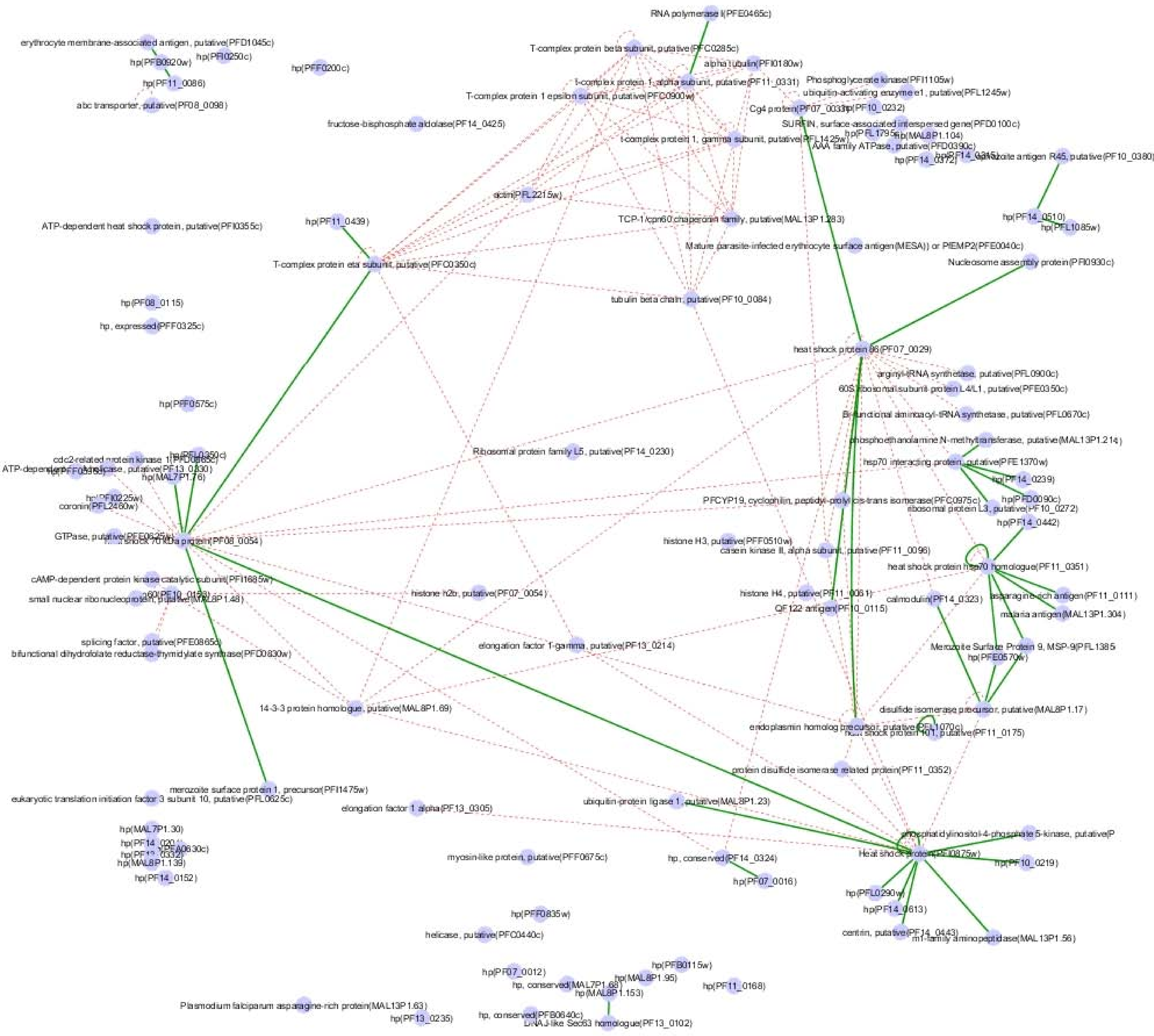

RNA polymerase II(PFE0465c)
erythrocyte membrane-associated antigen, putative(PFD1045c)
hp(PFB0920w)
hp(PFI0250c)
hp(PFF0200c)
hp(PF11_0086)
abc transporter, putative(PF08_0098)
T-complex protein beta subunit, putative(PFC0285c)
alpha tubulin(PFI0180w)
t-complex protein 1, alpha subunit, putative(PF11_0331)
T-complex protein 1 epsilon subunit, putative(PFC0900w)
Phosphoglycerate kinase(PFI1105w)
ubiquitin-activating enzyme e1, putative(PFL1245w)
Cg4 protein(PF07_0033)
hp(PF10_0232)
fructose-bisphosphate aldolase(PF14_0425)
SURFIN, surface-associated interspersed gene(PFD0100c)
hp(PFL1795c)
hp(MAL8P1.104)
t-complex protein 1, gamma subunit, putative(PFL1425w)
AAA family ATPase, putative(PFD0390c)
hp(PF14_0372)
merozoite antigen R45, putative(PF10_0380)
actin(PFL2215w)
ATP-dependent heat shock protein, putative(PFI0355c)
hp(PF11_0439)
TCP-1/cpn60 chaperonin family, putative(MAL13P1.283)
hp(PF14_0510)
hp(PFL1085w)
Mature parasite-infected erythrocyte surface antigen(MESA)) or PfEMP2(PFE0040c)
T-complex protein eta subunit, putative(PFC0350c)
Nucleosome assembly protein(PFI0930c)
hp(PF08_0115)
tubulin beta chain, putative(PF10_0084)
hp, expressed(PFF0325c)
heat shock protein 86(PF07_0029)
arginyl-tRNA synthetase, putative(PFL0900c)
60S ribosomal subunit protein L4/L1, putative(PFE0350c)
hp(PFF0575c)
Bifunctional aminoacyl-tRNA synthetase, putative(PFL0670c)
phosphoethanolamine N-methyltransferase, putative(MAL13P1.214)
cdc2-related protein kinase 1
Ribosomal protein family L5, putative(PF14_0230)
hsp70 interacting protein, putative(PFE1370w)
hp(MAL7P1.76)
hp(PF14_0239)
hp(PFI0225w)
PfCYP19, cyclophilin, peptidyl-prolyl cis-trans isomerase(PFC0975c)
hp(PFD0090c)
coronin(PFL2460w)
ribosomal protein L3, putative(PF10_0272)
hp(PF14_0442)
GTPase, putative
heat shock 70 kDa protein(PF08_0054)
histone H3, putative(PFF0510w)
casein kinase II, alpha subunit, putative(PF11_0096)
heat shock protein hsp70 homologue(PF11_0351)
cAMP-dependent protein kinase catalytic subunit(PFI1685w)
histone h2b, putative(PF07_0054)
histone H4, putative(PF11_0061)
calmodulin(PF14_0323)
asparagine-rich antigen(PF11_0111)
small nuclear ribonucleoprotein, putative(MAL8P1.48)
QF122 antigen(PF10_0115)
malaria antigen(MAL13P1.304)
splicing factor, putative(PFE0865c)
elongation factor 1-gamma, putative(PF13_0214)
Merozoite Surface Protein 9, MSP-9(PFL1385
bifunctional dihydrofolate reductase-thymidylate synthase(PFD0830w)
hp(PFE0570w)
14-3-3 protein homologue, putative(MAL8P1.69)
disulfide isomerase precursor, putative(MAL8P1.17)
endoplasmin homolog precursor, putative(PFL1070c)
heat shock protein 101, putative(PF11_0175)
protein disulfide isomerase related protein(PF11_0352)
merozoite surface protein 1, precursor(PFI1475w)
eukaryotic translation initiation factor 3 subunit 10, putative(PFL0625c)
elongation factor 1 alpha(PF13_0305)
ubiquitin-protein ligase 1, putative(MAL8P1.23)
hp(MAL7P1.30)
phosphatidylinositol-4-phosphate 5-kinase, putative(P
hp(MAL8P1.139)
myosin-like protein, putative(PFF0675c)
hp, conserved(PF14_0324)
Heat shock protein(PFI0875w)
hp(PF10_0219)
hp(PF14_0152)
hp(PF07_0016)
hp(PFL0290w)
hp(PF14_0613)
hp(PFF0835w)
helicase, putative(PFC0440c)
centrin, putative(PF14_0443)
m1-family aminopeptidase(MAL13P1.56)
hp(PFB0115w)
hp(PF07_0012)
hp(MAL8P1.95)
hp, conserved(MAL7P1.68)
hp(PF11_0168)
hp(MAL8P1.153)
Plasmodium falciparum asparagine-rich protein(MAL13P1.63)
hp, conserved(PFB0640c)
hp(PF13_0235)
DNAJ-like Sec63 homologue(PF13_0102)

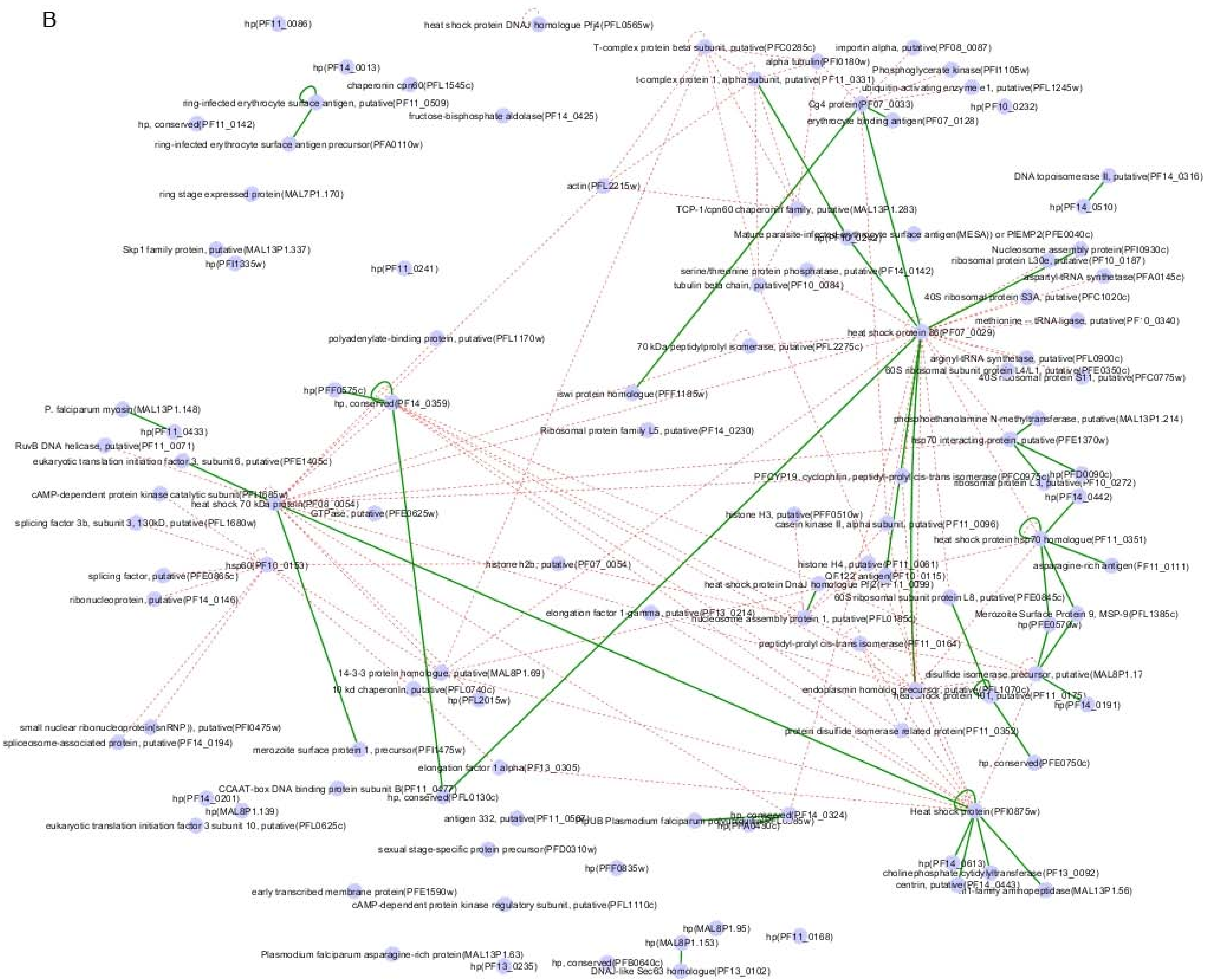
B
hp(PF11_0086)
heat shock protein DNAJ homologue Pfj4(PFL0565w)
T-complex protein beta subunit, putative(PFC0285c)
importin alpha, putative(PF08_0087)
alpha tubulin(PFI0180w)
Phosphoglycerate kinase(PFI1105w)
t-complex protein 1, alpha subunit, putative(PF11_0331)
ubiquitin-activating enzyme e1, putative(PFL1245w)
Cg4 protein(PF07_0033)
hp(PF10_0232)
erythrocyte binding antigen(PF07_0128)
hp(PF14_0013)
chaperonin cpn60(PFL1545c)
ring-infected erythrocyte surface antigen, putative(PF11_0509)
fructose-bisphosphate aldolase(PF14_0425)
hp, conserved(PF11_0142)
ring-infected erythrocyte surface antigen precursor(PFA0110w)
DNA topoisomerase II, putative(PF14_0316)
ring stage expressed protein(MAL7P1.170)
actin(PFL2215w)
hp(PF14_0510)
TCP-1/cpn60 chaperonin family, putative(MAL13P1.283)
Mature parasite-infected erythrocyte surface antigen(MESA)) or PfEMP2(PFE0040c)
hp(PF10_0242)
Skp1 family protein, putative(MAL13P1.337)
Nucleosome assembly protein(PFI0930c)
hp(PFI1335w)
ribosomal protein L30e, putative(PF10_0187)
hp(PF11_0241)
serine/threonine protein phosphatase, putative(PF14_0142)
aspartyl-tRNA synthetase(PFA0145c)
tubulin beta chain, putative(PF10_0084)
40S ribosomal protein S3A, putative(PFC1020c)
heat shock protein 86(PF07_0029)
methionine -- tRNA ligase, putative(PF10_0340)
polyadenylate-binding protein, putative(PFL1170w)
70 kDa peptidylprolyl isomerase, putative(PFL2275c)
arginyl-tRNA synthetase, putative(PFL0900c)
60S ribosomal subunit protein L4/L1, putative(PFE0350c)
40S ribosomal protein S11, putative(PFC0775w)
hp(PFF0575c)
hp, conserved(PF14_0359)
iswi protein homologue(PFF1185w)
P. falciparum myosin(MAL13P1.148)
phosphoethanolamine N-methyltransferase, putative(MAL13P1.214)
hp(PF11_0433)
Ribosomal protein family L5, putative(PF14_0230)
RuvB DNA helicase, putative(PF11_0071)
hsp70 interacting protein, putative(PFE1370w)
eukaryotic translation initiation factor 3, subunit 6, putative(PFE1405c)
PFCYP19, cyclophilin, peptidyl-prolyl cis-trans isomerase(PFC0975c)
hp(PFD0090c)
ribosomal protein L3, putative(PF10_0272)
cAMP-dependent protein kinase catalytic subunit(PFI1685w)
heat shock 70 kDa protein(PF08_0054)
hp(PF14_0442)
GTPase, putative(PFE0625w)
histone H3, putative(PFF0510w)
splicing factor 3b, subunit 3, 130kD, putative(PFL1680w)
casein kinase II, alpha subunit, putative(PF11_0096)
heat shock protein hsp70 homologue(PF11_0351)
hsp60(PF10_0153)
histone h2b, putative(PF07_0054)
histone H4, putative(PF11_0061)
asparagine-rich antigen(PF11_0111)
splicing factor, putative(PFE0865c)
QF122 antigen(PF10_0115)
heat shock protein DnaJ homologue Pfj2(PF11_0099)
ribonucleoprotein, putative(PF14_0146)
60S ribosomal subunit protein L8, putative(PFE0845c)
elongation factor 1-gamma, putative(PF13_0214)
nucleosome assembly protein 1, putative(PFL0185c)
Merozoite Surface Protein 9, MSP-9(PFL1385c)
hp(PFE0570w)
peptidyl-prolyl cis-trans isomerase(PF11_0164)
14-3-3 protein homologue, putative(MAL8P1.69)
disulfide isomerase precursor, putative(MAL8P1.17
10 kd chaperonin, putative(PFL0740c)
endoplasmin homolog precursor, putative(PFL1070c)
heat shock protein 101, putative(PF11_0175)
hp(PFL2015w)
hp(PF14_0191)
small nuclear ribonucleoprotein(snRNP), putative(PFI0475w)
protein disulfide isomerase related protein(PF11_0352)
spliceosome-associated protein, putative(PF14_0194)
merozoite surface protein 1, precursor(PFI1475w)
hp, conserved(PFE0750c)
elongation factor 1 alpha(PF13_0305)
CCAAT-box DNA binding protein subunit B(PF11_0477)
hp(PF14_0201)
hp, conserved(PFL0130c)
hp(MAL8P1.139)
Heat shock protein(PFI0875w)
antigen 332, putative(PF11_0507)
PfPUB Plasmodium falciparum polyubiquitin(PFL0585w)
hp, conserved(PF14_0324)
hp(PFA0430c)
eukaryotic translation initiation factor 3 subunit 10, putative(PFL0625c)
sexual stage-specific protein precursor(PFD0310w)
hp(PF14_0613)
hp(PFF0835w)
cholinephosphate cytidylyltransferase(PF13_0092)
centrin, putative(PF14_0443)
early transcribed membrane protein(PFE1590w)
M1-family aminopeptidase(MAL13P1.56)
cAMP-dependent protein kinase regulatory subunit, putative(PFL1110c)
hp(MAL8P1.95)
hp(PF11_0168)
hp(MAL8P1.153)
Plasmodium falciparum asparagine-rich protein(MAL13P1.63)
hp, conserved(PFB0640c)
hp(PF13_0235)
DNAJ-like Sec63 homologue(PF13_0102)

C

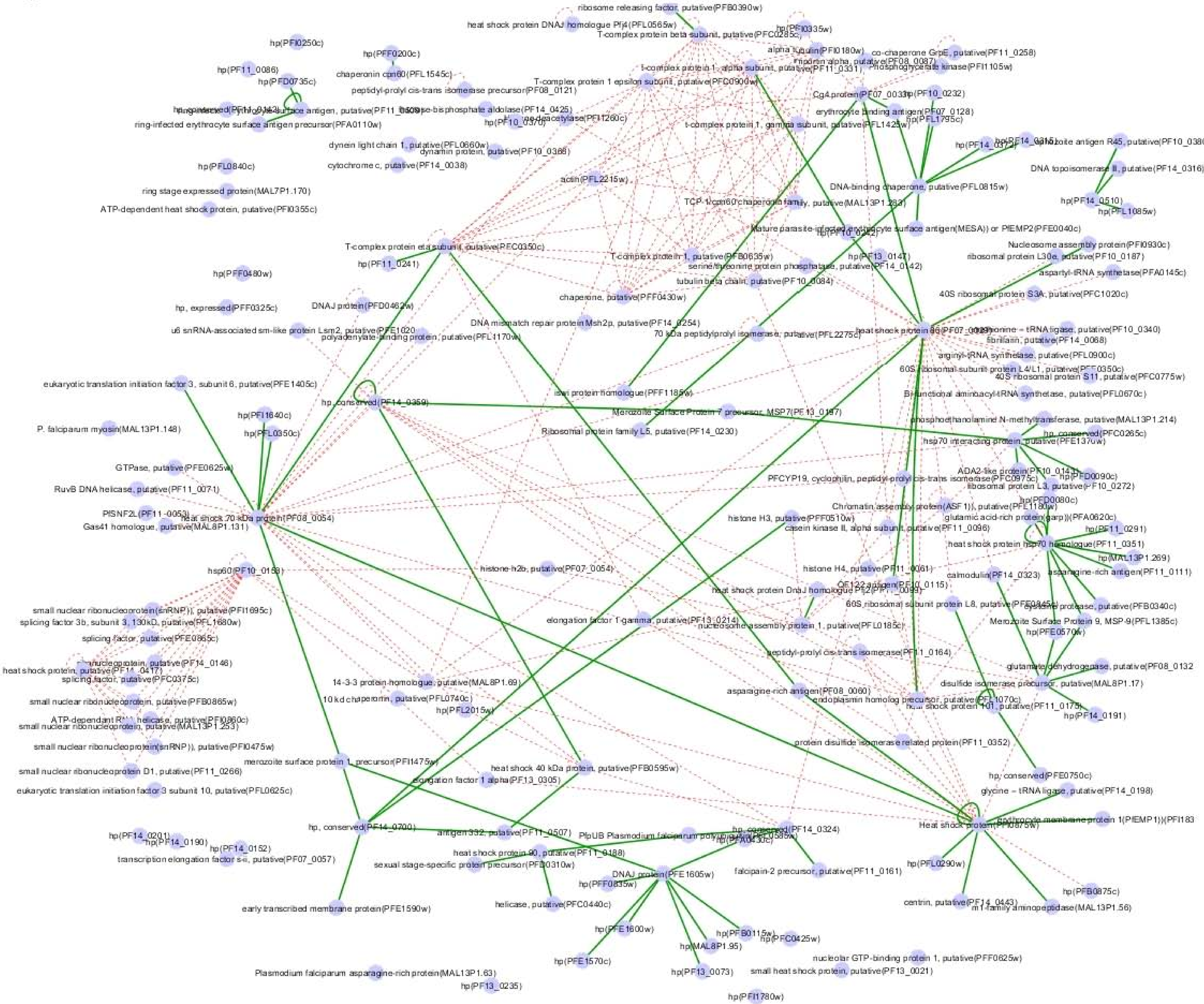

ribosome releasing factor, putative(PFB0390w)
heat shock protein DNAJ homologue Pfj4(PFL0565w)
T-complex protein beta subunit, putative(PFC0285c)
hp(PFI0335w)
hp(PFI0250c)
hp(PFF0200c)
alpha tubulin(PFI0180w)
co-chaperone GrpE, putative(PF11_0258)
hp(PF11_0086)
chaperonin cpn60(PFL1545c)
phosphoglycerate kinase(PFI1105w)
hp(PFD0735c)
T-complex protein 1 epsilon subunit, putative(PFC0900w)
peptidyl-prolyl cis-trans isomerase precursor(PF08_0121)
Cg4 protein(PF07_0033)
hp(PF10_0232)
erythrocyte binding antigen(PF07_0128)
ring-infected erythrocyte surface antigen precursor(PFA0110w)
hp(PF10_0370)
hp(PFL1795c)
t-complex protein 1, gamma subunit, putative(PFL1425w)
DNA topoisomerase II, putative(PF14_0316)
dynein light chain 1, putative(PFL0660w)
dynamin protein, putative(PF10_0368)
cytochrome c, putative(PF14_0038)
hp(PFL0840c)
actin(PFL2215w)
DNA-binding chaperone, putative(PFL0815w)
ring stage expressed protein(MAL7P1.170)
hp(PF14_0510)
ATP-dependent heat shock protein, putative(PFI0355c)
TCP-1/cpn60 chaperonin family, putative(MAL13P1.283)
hp(PFL1085w)
Nucleosome assembly protein(PFI0930c)
T-complex protein eta subunit, putative(PFC0350c)
ribosomal protein L30e, putative(PF10_0187)
hp(PF11_0241)
T-complex protein 1, putative(PFB0635w)
hp(PF13_0147)
serine/threonine protein phosphatase, putative(PF14_0142)
hp(PFF0480w)
aspartyl-tRNA synthetase(PFA0145c)
tubulin beta chain, putative(PF10_0084)
chaperone, putative(PFF0430w)
40S ribosomal protein S3A, putative(PFC1020c)
hp, expressed(PFF0325c)
DNAJ protein(PFD0462w)
DNA mismatch repair protein Msh2p, putative(PF14_0254)
70 kDa peptidylprolyl isomerase, putative(PFL2275c)
fibrillarin, putative(PF14_0068)
polyadenylate-binding protein, putative(PFL1170w)
arginyl-tRNA synthetase, putative(PFL0900c)
40S ribosomal protein S11, putative(PFC0775w)
eukaryotic translation initiation factor 3, subunit 6, putative(PFE1405c)
iswi protein homologue(PFF1185w)
Bi-functional aminoacyl-tRNA synthetase, putative(PFL0670c)
hp, conserved(PF14_0359)
hp(PFI1640c)
Merozoite Surface Protein 7 precursor, MSP7(PF13_0197)
phosphoethanolamine N-methyltransferase, putative(MAL13P1.214)
P. falciparum myosin(MAL13P1.148)
hp(PFL0350c)
Ribosomal protein family L5, putative(PF14_0230)
hp, conserved(PFC0265c)
hsp70 interacting protein, putative(PFE1370w)
GTPase, putative(PFE0625w)
hp(PFD0090c)
RuvB DNA helicase, putative(PF11_0071)
PFCYP19, cyclophilin, peptidyl-prolyl cis-trans isomerase(PFC0975c)
ribosomal protein L3, putative(PF10_0272)
hp(PFD0080c)
Gas41 homologue, putative(MAL8P1.131)
histone H3, putative(PFF0510w)
casein kinase II, alpha subunit, putative(PF11_0096)
hp(PF11_0291)
heat shock protein hsp70 homologue(PF11_0351)
hp(MAL13P1.269)
hsp60(PF10_0153)
histone-h2b, putative(PF07_0054)
histone H4, putative(PF11_0061)
calmodulin(PF14_0323)
asparagine-rich antigen(PF11_0111)
small nuclear ribonucleoprotein(snRNP)), putative(PFI1695c)
splicing factor 3b, subunit 3, 130kD, putative(PFL1680w)
elongation factor 1-gamma, putative(PF13_0214)
nucleosome assembly protein 1, putative(PFL0185c)
cysteine protease, putative(PFB0340c)
Merozoite Surface Protein 9, MSP-9(PFL1385c)
splicing factor, putative(PFE0865c)
hp(PFE0570w)
peptidyl-prolyl cis-trans isomerase(PF11_0164)
glutamate dehydrogenase, putative(PF08_0132)
splicing factor, putative(PFC0375c)
14-3-3 protein homologue, putative(MAL8P1.69)
disulfide isomerase precursor, putative(MAL8P1.17)
10 kd chaperonin, putative(PFL0740c)
asparagine-rich antigen(PF08_0060)
endoplasmin homolog precursor, putative(PFL1070c)
small nuclear ribonucleoprotein, putative(PFB0865w)
hp(PFL2015w)
hp(PF14_0191)
small nuclear ribonucleoprotein, putative(MAL13P1.253)
small nuclear ribonucleoprotein(snRNP)), putative(PFI0475w)
protein disulfide isomerase related protein(PF11_0352)
merozoite surface protein 1, precursor(PFI1475w)
heat shock 40 kDa protein, putative(PFB0595w)
small nuclear ribonucleoprotein D1, putative(PF11_0266)
hp, conserved(PFE0750c)
elongation factor 1 alpha(PF13_0305)
glycine - tRNA ligase, putative(PF14_0198)
eukaryotic translation initiation factor 3 subunit 10, putative(PFL0625c)
hp, conserved(PF14_0700)
hp, conserved(PF14_0324)
hp(PF14_0201)
hp(PF14_0190)
antigen 332, putative(PF11_0507)
hp(PF14_0152)
heat shock protein 90, putative(PF11_0188)
transcription elongation factor s-ii, putative(PF07_0057)
hp(PFL0290w)
sexual stage-specific protein precursor(PFD0310w)
DNAJ protein(PFE1605w)
falcipain-2 precursor, putative(PF11_0161)
hp(PFF0835w)
hp(PFB0875c)
helicase, putative(PFC0440c)
early transcribed membrane protein(PFE1590w)
m1-family aminopeptidase(MAL13P1.56)
hp(PFE1600w)
hp(PFB0115w)
hp(PFC0425w)
hp(MAL8P1.95)
hp(PFE1570c)
nucleolar GTP-binding protein 1, putative(PFF0625w)
hp(PF13_0073)
small heat shock protein, putative(PF13_0021)
Plasmodium falciparum asparagine-rich protein(MAL13P1.63)
hp(PF13_0235)
hp(PFI1780w)

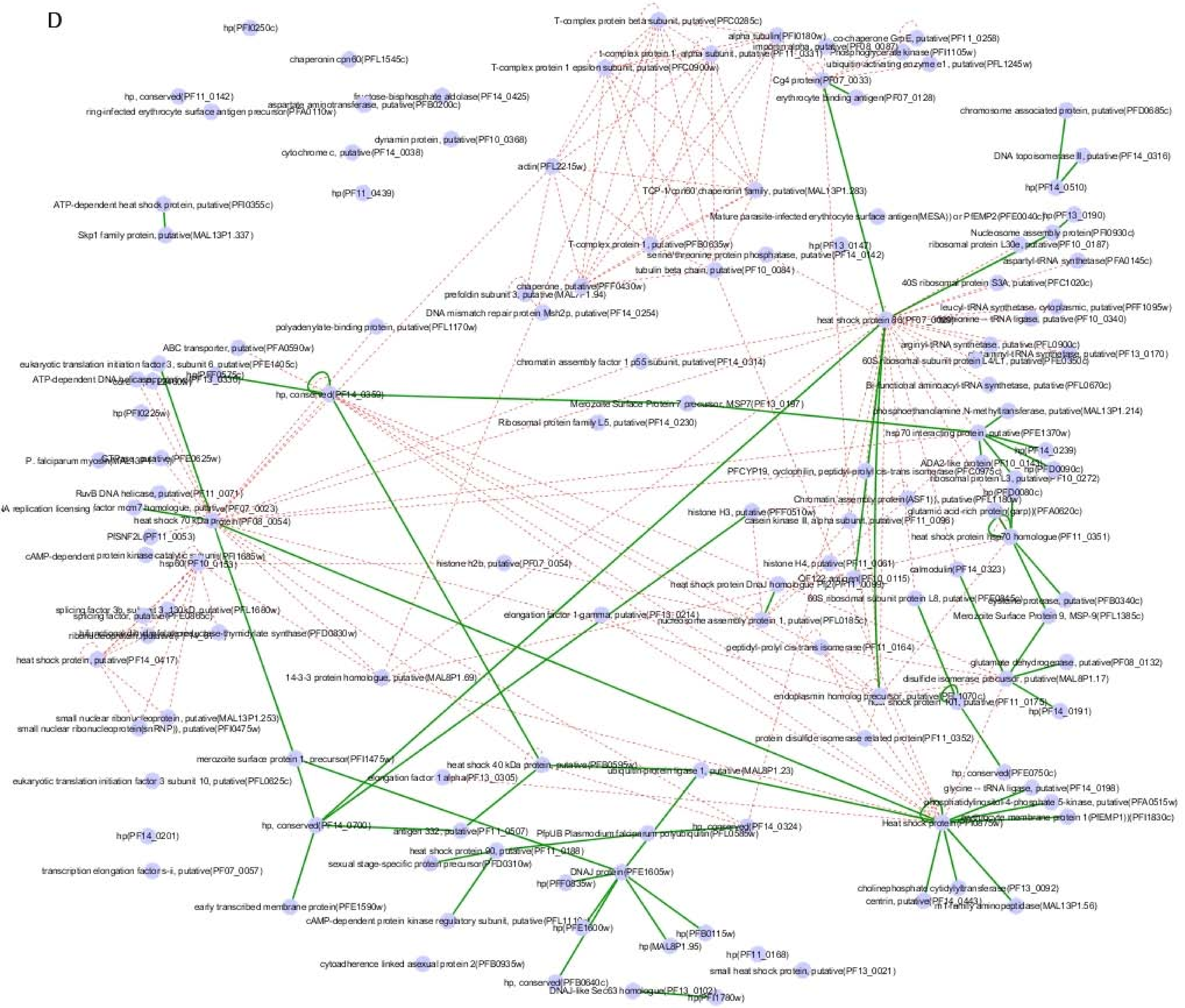
D
hp(PFI0250c)
chaperonin cpn60(PFL1545c)
hp, conserved(PF11_0142)
ring-infected erythrocyte surface antigen precursor(PFA0110w)
fructose-bisphosphate aldolase(PF14_0425)
aspartate aminotransferase, putative(PFB0200c)
dynamin protein, putative(PF10_0368)
cytochrome c, putative(PF14_0038)
hp(PF11_0439)
ATP-dependent heat shock protein, putative(PFI0355c)
Skp1 family protein, putative(MAL13P1.337)
T-complex protein beta subunit, putative(PFC0285c)
alpha tubulin(PFI0180w)
co-chaperone GrpE, putative(PF11_0258)
Phosphoglycerate kinase(PFI1105w)
T-complex protein 1 epsilon subunit, putative(PFC0900w)
ubiquitin-activating enzyme e1, putative(PFL1245w)
Cg4 protein(PF07_0033)
erythrocyte binding antigen(PF07_0128)
chromosome associated protein, putative(PFD0685c)
DNA topoisomerase II, putative(PF14_0316)
hp(PF14_0510)
actin(PFL2215w)
TCP-1/cpn60 chaperonin family, putative(MAL13P1.283)
Mature parasite-infected erythrocyte surface antigen(MESA)) or PfEMP2(PFE0040c)
hp(PF13_0190)
Nucleosome assembly protein(PFI0930c)
ribosomal protein L30e, putative(PF10_0187)
aspartyl-tRNA synthetase(PFA0145c)
T-complex protein-1, putative(PFB0635w)
hp(PF13_0147)
serine/threonine protein phosphatase, putative(PF14_0142)
tubulin beta chain, putative(PF10_0084)
40S ribosomal protein S3A, putative(PFC1020c)
chaperone, putative(PFF0430w)
leucyl-tRNA synthetase, cytoplasmic, putative(PFF1095w)
DNA mismatch repair protein Msh2p, putative(PF14_0254)
heat shock protein 86(PF07_0029)
polyadenylate-binding protein, putative(PFL1170w)
arginyl-tRNA synthetase, putative(PFL0900c)
ABC transporter, putative(PFA0590w)
eukaryotic translation initiation factor 3, subunit 6, putative(PFE1405c)
chromatin assembly factor 1 p55 subunit, putative(PF14_0314)
Bi-functional aminoacyl-tRNA synthetase, putative(PFL0670c)
hp, conserved(PF14_0359)
Merozoite Surface Protein 7 precursor, MSP7(PF13_0197)
phosphoethanolamine N-methyltransferase, putative(MAL13P1.214)
hp(PFI0225w)
Ribosomal protein family L5, putative(PF14_0230)
hsp70 interacting protein, putative(PFE1370w)
hp(PF14_0239)
hp(PFD0090c)
hp(PFD0080c)
RuvB DNA helicase, putative(PF11_0071)
heat shock 70 kDa protein(PF08_0054)
PfSNF2L(PF11_0053)
hsp60(PF10_0153)
histone H3, putative(PFF0510w)
casein kinase II, alpha subunit, putative(PF11_0096)
glutamic acid-rich protein(garp))(PFA0620c)
heat shock protein hsp70 homologue(PF11_0351)
calmodulin(PF14_0323)
histone h2b, putative(PF07_0054)
histone H4, putative(PF11_0061)
QF122 antigen(PF10_0115)
elongation factor 1-gamma, putative(PF13_0214)
nucleosome assembly protein 1, putative(PFL0185c)
cysteine protease, putative(PFB0340c)
Merozoite Surface Protein 9, MSP-9(PFL1385c)
heat shock protein, putative(PF14_0417)
14-3-3 protein homologue, putative(MAL8P1.69)
peptidyl-prolyl cis-trans isomerase(PF11_0164)
glutamate dehydrogenase, putative(PF08_0132)
disulfide isomerase precursor, putative(MAL8P1.17)
endoplasmin homolog precursor, putative(PFL1070c)
heat shock protein 101, putative(PF11_0175)
hp(PF14_0191)
small nuclear ribonucleoprotein, putative(MAL13P1.253)
small nuclear ribonucleoprotein(snRNP)), putative(PFI0475w)
protein disulfide isomerase related protein(PF11_0352)
merozoite surface protein 1, precursor(PFI1475w)
heat shock 40 kDa protein, putative(PFB0595w)
ubiquitin-protein ligase 1, putative(MAL8P1.23)
eukaryotic translation initiation factor 3 subunit 10, putative(PFL0625c)
elongation factor 1 alpha(PF13_0305)
hp, conserved(PFE0750c)
glycine -- tRNA ligase, putative(PF14_0198)
phosphatidylinositol-4-phosphate 5-kinase, putative(PFA0515w)
hp(PF14_0201)
hp, conserved(PF14_0700)
antigen 332, putative(PF11_0507)
PfpUB Plasmodium falciparum polyubiquitin(PFL0585w)
hp, conserved(PF14_0324)
Heat shock protein(PFI0875w)
erythrocyte membrane protein 1(PfEMP1))(PFI1830c)
heat shock protein 90, putative(PF11_0188)
sexual stage-specific protein precursor(PFD0310w)
transcription elongation factor s-ii, putative(PF07_0057)
DNAJ protein(PFE1605w)
hp(PFF0835w)
cholinephosphate cytidylyltransferase(PF13_0092)
centrin, putative(PF14_0443)
early transcribed membrane protein(PFE1590w)
hp(PFE1600w)
hp(PFB0115w)
hp(MAL8P1.95)
hp(PF11_0168)
cytoadherence linked asexual protein 2(PFB0935w)
small heat shock protein, putative(PF13_0021)
hp, conserved(PFB0640c)
DNAJ-like Sec63 homologue(PF13_0102)
hp(PFI1780w)

E

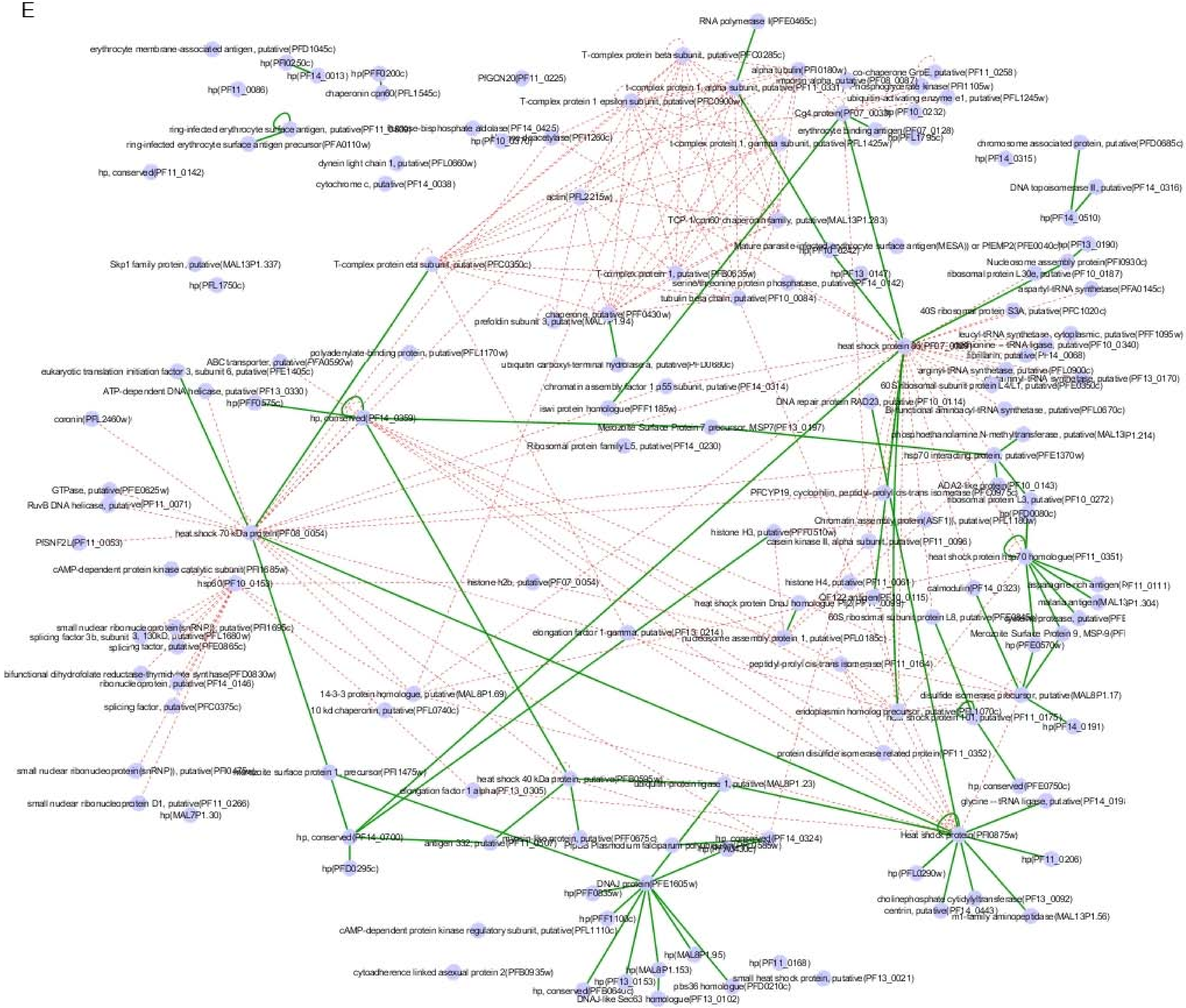

RNA polymerase II(PFE0465c)
erythrocyte membrane-associated antigen, putative(PFD1045c)
hp(PFI0250c)
hp(PF14_0013)
hp(PFF0200c)
chaperonin cpn60(PFL1545c)
hp(PF11_0086)
PfGCN20(PF11_0225)
T-complex protein beta subunit, putative(PFC0285c)
alpha tubulin(PFI0180w)
co-chaperone GrpE, putative(PF11_0258)
ubiquitin-activating enzyme e1, putative(PFL1245w)
T-complex protein 1 epsilon subunit, putative(PFC0900w)
Cg4 protein(PF07_0033)
hp(PF10_0232)
ring-infected erythrocyte surface antigen precursor(PFA0110w)
histone deacetylase(PFI1260c)
erythrocyte binding antigen(PF07_0128)
hp(PFL1795c)
t-complex protein 1, gamma subunit, putative(PFL1425w)
chromosome associated protein, putative(PFD0685c)
hp(PF14_0315)
dynein light chain 1, putative(PFL0660w)
hp, conserved(PF11_0142)
cytochrome c, putative(PF14_0038)
DNA topoisomerase II, putative(PF14_0316)
actin(PFL2215w)
hp(PF14_0510)
TCP-1/cpn60 chaperonin family, putative(MAL13P1.283)
hp(PF13_0190)
Skp1 family protein, putative(MAL13P1.337)
T-complex protein eta subunit, putative(PFC0350c)
Nucleosome assembly protein(PFI0930c)
hp(PFL1750c)
T-complex protein 1, putative(PFB0635w)
hp(PF13_0147)
ribosomal protein L30e, putative(PF10_0187)
aspartyl-tRNA synthetase(PFA0145c)
tubulin beta chain, putative(PF10_0084)
40S ribosomal protein S3A, putative(PFC1020c)
chaperone, putative(PFF0430w)
prefoldin subunit 3, putative
leucyl-tRNA synthetase, cytoplasmic, putative(PFF1095w)
heat shock protein 90(PF07_0029)
polyadenylate-binding protein, putative(PFL1170w)
ABC transporter, putative(PFA0590w)
eukaryotic translation initiation factor 3, subunit 6, putative(PFE1405c)
arginyl-tRNA synthetase, putative(PFL0900c)
ATP-dependent DNA helicase, putative(PF13_0330)
chromatin assembly factor 1 p55 subunit, putative(PF14_0314)
hp(PFF0575c)
iswi protein homologue(PFF1185w)
DNA repair protein RAD23, putative(PF10_0114)
coronin(PFL2460w)
hp, conserved(PF14_0359)
Bifunctional aminoacyl-tRNA synthetase, putative(PFL0670c)
Merozoite Surface Protein 7 precursor, MSP7(PF13_0197)
phosphoethanolamine N-methyltransferase, putative(MAL13P1.214)
Ribosomal protein family L5, putative(PF14_0230)
hsp70 interacting protein, putative(PFE1370w)
ADA2-like protein(PF10_0143)
PfCYP19, cyclophilin, peptidyl-prolyl cis-trans isomerase
GTPase, putative(PFE0625w)
ribosomal protein L3, putative(PF10_0272)
RuvB DNA helicase, putative(PF11_0071)
hp(PFD0080c)
Chromatin assembly protein(ASF1), putative(PFL1180w)
heat shock 70 kDa protein(PF08_0054)
histone H3, putative(PFF0510w)
PfSNF2L(PF11_0053)
casein kinase II, alpha subunit, putative(PF11_0096)
heat shock protein hsp70 homologue(PF11_0351)
cAMP-dependent protein kinase catalytic subunit(PFI1685w)
hsp60(PF10_0153)
histone-h2b, putative(PF07_0054)
histone H4, putative(PF11_0061)
calmodulin(PF14_0323)
QF122 antigen(PF10_0115)
heat shock protein DnaJ homologue
malaria antigen(MAL13P1.304)
small nuclear ribonucleoprotein(snRNP), putative(PFI1695c)
splicing factor 3b, subunit 3, 130kD, putative(PFL1680w)
60S ribosomal subunit protein L8, putative
splicing factor, putative(PFE0865c)
nucleosome assembly protein 1, putative(PFL0185c)
elongation factor 1-gamma, putative(PF13_0214)
hp(PFE0570w)
bifunctional dihydrofolate reductase-thymidylate synthase(PFD0830w)
ribonucleoprotein, putative(PF14_0146)
peptidyl-prolyl cis-trans isomerase(PF11_0164)
14-3-3 protein homologue, putative(MAL8P1.69)
disulfide isomerase precursor, putative(MAL8P1.17)
splicing factor, putative(PFC0375c)
10 kd chaperonin, putative(PFL0740c)
endoplasmin homolog precursor, putative(PFL1070c)
hp(PF14_0191)
protein disulfide isomerase related protein(PF11_0352)
small nuclear ribonucleoprotein D1, putative(PF11_0266)
heat shock 40 kDa protein, putative(PFB0595w)
ubiquitin protein ligase 1, putative(MAL8P1.23)
elongation factor 1 alpha(PF13_0305)
hp, conserved(PFE0750c)
hp(MAL7P1.30)
glycine -- tRNA ligase, putative
hp, conserved(PF14_0700)
hp, conserved(PF14_0324)
Heat shock protein(PFI0875w)
hp(PFD0295c)
hp(PF11_0206)
hp(PFL0290w)
DNAJ protein(PFE1605w)
hp(PFF0835w)
cholinephosphate cytidylyltransferase(PF13_0092)
centrin, putative(PF14_0443)
m1-family aminopeptidase(MAL13P1.56)
hp(PFF1100c)
cAMP-dependent protein kinase regulatory subunit, putative(PFL1110c)
hp(MAL8P1.95)
hp(PF11_0168)
hp(MAL8P1.153)
cytoadherence linked asexual protein 2(PFB0935w)
small heat shock protein, putative(PF13_0021)
hp(PF13_0153)
pbs36 homologue(PFD0210c)
DNAJ-like Sec63 homologue(PF13_0102)
